# Wafer-Scale Characterization of 1692-Pixel-Per-Inch Blue Micro-LED Arrays with an Optimized ITO Layer

**DOI:** 10.3390/mi15050560

**Published:** 2024-04-24

**Authors:** Eun-Kyung Chu, Eun Jeong Youn, Hyun Woong Kim, Bum Doo Park, Ho Kun Sung, Hyeong-Ho Park

**Affiliations:** 1 Optical Device Laboratory, Korea Advanced Nano Fab Center (KANC), Suwon 443270, Republic of Korea; eunkyung.chu@kanc.re.kr (E.-K.C.); eunjeong.youn@kanc.re.kr (E.J.Y.); 2 Convergence Technology Division, Korea Advanced Nano Fab Center (KANC), Suwon 443270, Republic of Korea; hyunwoong.kim@kanc.re.kr (H.W.K.); bumdoo.park@kanc.re.kr (B.D.P.); hokun.sung@kanc.re.kr (H.K.S.)

**Keywords:** micro-light-emitting diode, indium tin oxide, wafer-scale characterization, high pixel-per-inch

## Abstract

Wafer-scale blue micro-light-emitting diode (micro-LED) arrays were fabricated with a pixel size of 12 μm, a pixel pitch of 15 μm, and a pixel density of 1692 pixels per inch, achieved by optimizing the properties of e-beam-deposited and sputter-deposited indium tin oxide (ITO). Although the sputter-deposited ITO (S-ITO) films exhibited a densely packed morphology and lower resistivity compared to the e-beam-deposited ITO (E-ITO) films, the forward voltage (V_F_) values of a micro-LED with the S-ITO films were higher than those with the E-ITO films. The V_F_ values for a single pixel and for four pixels with E-ITO films were 2.82 V and 2.83 V, respectively, while the corresponding values for S-ITO films were 3.50 V and 3.52 V. This was attributed to ion bombardment damage and nitrogen vacancies in the p-GaN layer. Surprisingly, the V_F_ variations of a single pixel and of four pixels with the optimized E-ITO spreading layer from five different regions were only 0.09 V and 0.10 V, respectively. This extremely uniform V_F_ variation is suitable for creating micro-LED displays to be used in AR and VR applications, circumventing the bottleneck in the development of long-lifespan and high-brightness organic LED devices for industrial mass production.

## 1. Introduction

Micro-light-emitting diode (micro-LED) technology continues to attract strong interest due to the high resolutions, outstanding luminous efficiency, remarkable brightness, and impressive durability that it can offer. These features make these types of diodes a most promising platform in high-end display applications such as mobile phones, wearable watches, and augmented reality (AR)/virtual reality (VR) displays, which require high luminance, high refresh rates, and high pixel-per-inch (PPI) values [1,2,3,4,5,6]. Specifically, displays beyond eye-limiting resolutions have attracted substantial interest as a key enabler of AR/VR displays [7,8]. Given that next-generation displays require high optical contrast levels, sub-12 μm pixels must be used to achieve highly saturated images and flicker-free images with minimal screen-door effects. This enhances the overall clarity of the image and alleviates eye strain [9,10]. Also, for application to near-eye displays, the resolution should exceed 1500 PPI. A high-resolution display provides enhanced immersion, a diminished screen-door effect, a sharply projected enlarged image, and heightened visual comfort during use [11].

With the increasing demand for micro-LEDs at present, the need for optimization research on indium tin oxide (ITO)—considered to be a promising electrode for the p-type GaN layer, among various current-spreading layers—has also increased [12,13,14,15]. ITO films with high transparency, low resistivity, and the ability to form reliable ohmic contacts with p-GaN are highly suitable for use as extended electrodes in micro-LED applications [16]. There are various deposition methods that can be used to obtain high-quality ITO films, such as electron-beam (e-beam) evaporation [17,18,19], magnetron sputtering [20,21], pulsed laser deposition (PLD) [22,23], sol–gel methods [24,25], and spray pyrolysis [26,27]. Among these, e-beam evaporation and sputtering techniques are widely used for ITO film deposition [28]. Additionally, magnetron sputtering presents distinct advantages, including high deposition rates, low deposition pressures, the production of high-quality films, enhanced adhesion, and superior uniformity across expansive surfaces [29]. The deposition method used for ITO can affect the surface roughness and electrical properties of the film. ITO optimization based on the deposition method is crucial for achieving high-resolution micro-LED arrays, especially those with high pixel densities and those capable of operating at high current densities.

To date, there has been extensive research and development on size-dependent effects of micro-LED arrays for display applications. J. Nie et al. reported that the notorious efficiency reduction induced by sidewall defects in small micro-LED arrays could be significantly reduced by applying short-pulse voltages [30]. Z. Wang et al. reported guidance for the analysis and repair of sidewall defects to improve the quantum efficiency of micro-LEDs’ display [31]. Also, K. Y. Yoo et al. reported that more accurate prediction of the chip-size-dependent efficiency of an LED wafer can be provided, allowing us to wisely evaluate proper wafers emitting various wavelengths for micro-LED display applications [32]. Despite the advancements in micro-LED technologies, several issues hinder their widespread application. For example, the application of near-eye displays to industrial mass production requires comprehensive wafer-scale characterization of blue micro-LED arrays [33]. To the best of our knowledge, the wafer-scale characterization of blue micro-LED arrays has not yet been reported in detail. In addition, we still need to improve the optimization of ITO films. This is associated with high power consumption resulting from the high forward voltage, attributed to the weakened optical and electrical properties of the ITO films [34]. These motivations drove us to undertake the four-inch wafer-scale characterization of high-performance blue micro-LED arrays with a resolution of 1692 PPI to obtain micro-LED displays with a high-density resolution.

This study presents the four-inch wafer-scale fabrication of blue micro-LED arrays on a sapphire substrate with a resolution of 1692 PPI, accomplished by optimizing the properties of e-beam-deposited and sputter-deposited ITO as a current-spreading layer to ultimately obtain high-performance micro-LED displays.

## 2. Experimental Section

Wafer-scale blue micro-LED arrays were fabricated with a pixel size of 12 μm and a pixel pitch of 15 μm, reaching 1692 PPI. InGaN/GaN-based epilayer structures were grown by metal–organic chemical vapor deposition (MOCVD) on a sapphire substrate. The InGaN/GaN-based epilayer structure was composed of a four-micrometer-thick undoped GaN layer, a 2.5-micrometer-thick Si-doped n-GaN layer, ten-period InGaN/GaN multi-quantum wells (MQWs), a 20-nanometer-thick p-AlGaN electron-blocking layer, and a 200-nanometer-thick Mg-doped p-GaN layer.

Prior to ITO deposition, the sample was dipped in sulfuric acid hydrogen peroxide solution (H_2_SO_4_:H_2_O_2_ = 4:1) for 15 min and then rinsed in running deionized water for 2 min to clean the p-GaN surface. The metallic ions and native oxides were removed from the p-GaN surface by immersing the sample in a HCl:H_2_O = 1:10 solution for one minute, followed by rinsing in running deionized water for 2 min. Afterwards, 200-nanometer-thick ITO films were deposited using an e-beam evaporator under a vacuum pressure of 7 × 10^−7^ Torr and a deposition rate of 0.05 nm/s, or by direct-current (DC) sputtering as the ohmic contact layer of a p-type GaN. The sputtering deposition was carried out in a system initially evacuated at 1 × 10^−5^ Torr, with a working pressure of 10^−3^ Torr in a pure argon atmosphere and a deposition rate of 0.3 nm/s. The composition of the ITO samples using the e-beam evaporator and DC sputtering was 10 wt% SnO_2_ and 90 wt% In_2_O_3_, respectively. Then, the mesa-structure was formed by inductively coupled plasma–reactive ion etching (ICP-RIE), reaching a pixel size of 12 µm × 12 µm. The etching conditions were 30 sccm of Cl_2_, 5 sccm of BCl_3_, and 5 mTorr total pressure. The GaN etching rate was approximately 0.24 μm/min, and the etch rates were measured using a depth profiler (α-step 500, KLA tencor). Subsequently, the sample was placed in a HCl:H_2_O = 1:10 solution for one minute to minimize the damage to the dry-etched sidewalls of the pixels. To ensure proper ohmic contact, the sample was annealed at 600 °C for 120 s in a N_2_ atmosphere by rapid thermal annealing (RTA). The RTA process used in this study comprised three stages: (i) the temperature was increased to the desired value within 30 s, (ii) the desired temperature was maintained for 120 s, and (iii) rapid cooling. Then, multilayer deposition with Cr (20 nm)/Au (500 nm) onto the ITO and the n-GaN layer was conducted to form individual p-electrodes and a common n-electrode, respectively. Also, the mesa-sidewall of each micro-LED pixel was passivated by a 500-nanometer-thick SiNx layer using chemical vapor deposition (CVD) to reduce the leakage current. To drive the passive matrix (PM)-type blue micro-LED arrays, the SiNx passivation layer was selectively etched using a buffer oxide etchant (BOE) until the individual p-electrodes and common n-electrode were exposed, after which Cr (20 nm) and Au (500 nm) p-pad and n-pad line layers were deposited, respectively. Because the corresponding pixel size and pixel pitch were 12 and 15 μm, respectively, the formed p-pad line layer passed over the pixels, with the p-electrodes of each micro-LED then selectively connected in a row for matrix-addressable driving. Samples labeled “E-ITO” were ITO films deposited by the e-beam evaporator, while samples termed “S-ITO” were ITO films deposited by sputtering.

**Figure 1:**
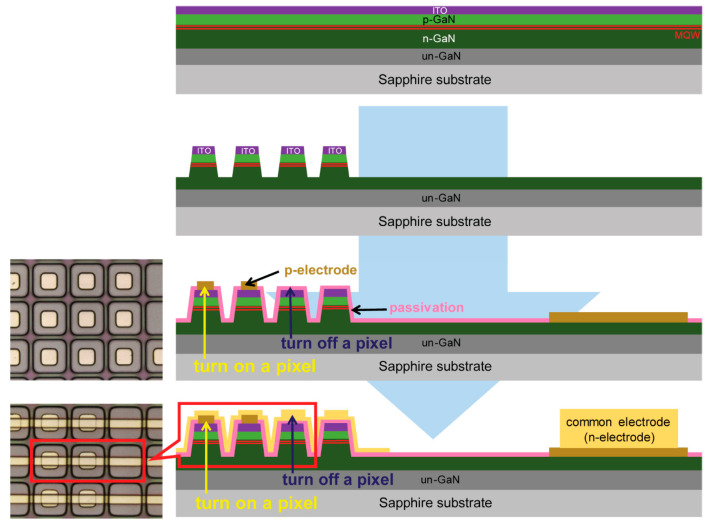
Schematic diagram of the fabrication process used to create the blue micro-LED arrays.

To produce various light-emitting images for the fabricated micro-LED arrays with hundreds of individual pixels, PM-type micro-LED arrays were achieved, as shown in Figure 1. According to the selective formation of the p-electrodes for the micro-LED arrays, each pixel could be individually turned on and off to demonstrate various monochrome images with a resolution of 1692 PPI. While a pixel with a p-electrode is a light-emitting pixel, a pixel without a p-electrode does not emit light.

The surface morphologies of the ITO films were studied using field-emission scanning electron microscopy (FE-SEM) (S-4800, Hitachi Ltd., Tokyo, Japan) and atomic force microscopy (AFM) (XE100, Park Systems, Suwon, Gyeonggi, Republic of Korea). Current density–voltage (J–V) measurements of the LEDs were obtained with a parameter analyzer (4200-SCS, Keithley Instruments, Cleveland, OH, USA). Wafer-scale characterization of the electroluminescence (EL) and optical output power of the blue micro-LEDs was conducted using a semi-auto LED prober at normal incidence (WPS3100, Opto System, Tokyo, Japan).

## 3. Results and Discussion

**Figure 2:**
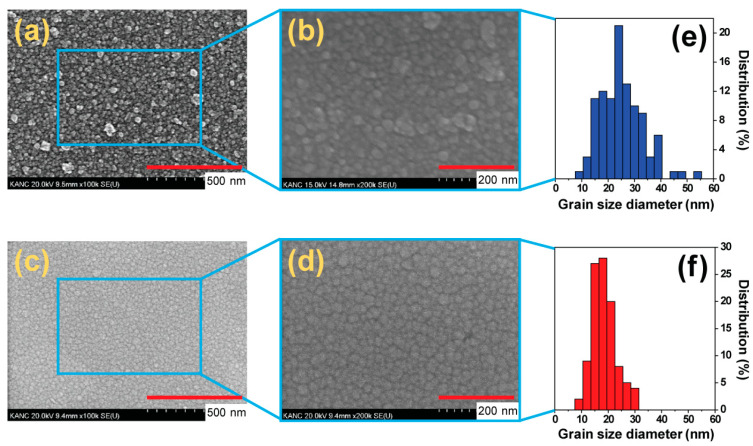
Top-view SEM images of E-ITO films: ((**a**) at 100,000× and (**b**) at 200,000×) and S-ITO films ((**c**) at 100,000× and (**d**) at 200,000×), and histograms showing the grain size distributions of (**b**) for (**e**) the E-ITO films and (**d**) for (**f**) the S-ITO films.

Quantitative analyses were conducted to determine the grain size distributions in the different ITO films deposited by e-beam evaporation and sputtering. The surface morphology and the grain size of the ITO films were measured by examining top-view SEM images, as shown in Figure 2a–d. The surface morphology of the S-ITO films was relatively smooth and dense, while that of the E-ITO films was rather rough. Grain size distribution histograms of the E-ITO films and the S-ITO films are shown in Figure 2e,f, respectively. The grain size distribution of the E-ITO films was found to have a broad size range, from 9 to 54 nm, while that of the S-ITO films showed a much narrower range (9–30 nm). The grain size distribution of the S-ITO films was significantly narrower compared to that of the E-ITO films. In addition, the average grain size of the E-ITO films was measured and found to be 24.8 nm, whereas that of the S-ITO films was 18.2 nm. The average grain size of the E-ITO films showed an incremental trend compared to that of the S-ITO films. In general, when particles are deposited at a high energy level with a substantial amount of migration present, the resulting grain size tends to be smaller. Conversely, if there is reduced migration during particle condensation on the substrate, the grain size will be larger [35]. In our case, in that sputtering is a relatively high-energy process, the S-ITO films showed a much narrower size distribution compared to the E-ITO films.

**Figure 3:**
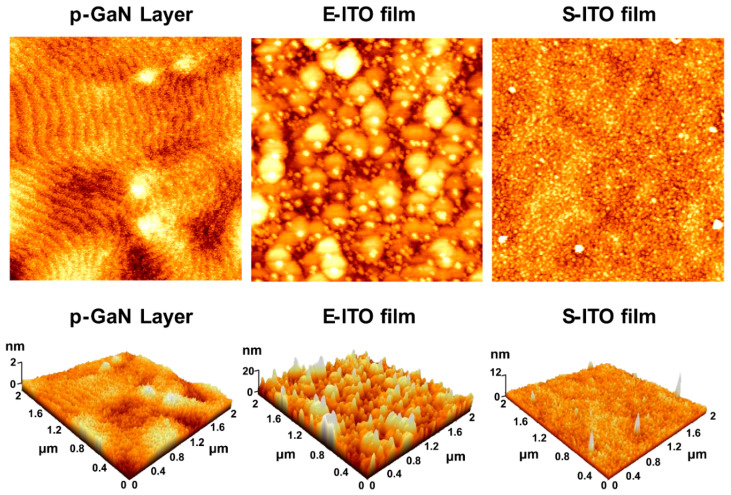
Two-dimensional (2D) and three-dimensional (3D) AFM images of a p-GaN layer, and 2D and 3D AFM images of E-ITO film and S-ITO film deposited onto a p-GaN layer.

The surface morphologies of the p-GaN layer and the ITO films deposited on the p-GaN layer with the two different deposition methods were observed by AFM; these results are shown in Figure 3. The measured RMS roughness values of the p-GaN layer, the E-ITO films, and the S-ITO films were 0.175, 5.187, and 0.824 nm, respectively. To investigate only the surface morphology of ITO films processed with the two different deposition methods, the films were calculated to eliminate the influence of the roughness of the sub-layer, in this case the p-GaN layer. The S-ITO films had a lower RMS roughness value of 0.649 nm compared to that of the E-ITO films, which had a RMS roughness value of 5.012 nm. The roughness of the E-ITO films was approximately 7.72 times higher than that of the S-ITO films. The S-ITO films exhibited smooth surfaces and had small grains, while the E-ITO films had grains with increased sizes, creating a rougher morphology. The difference in the surface morphology could be reflected in the corresponding film resistivity. The measured resistivity of the S-ITO films was 4.86 × 10^−4^ ohm·cm, much lower than that of the E-ITO films (5.96 × 10^−3^ ohm·cm). The surface AFM observations suggested that the different ITO films, deposited by the e-beam evaporation and sputtering, had distinct effects on the resistivity as well as the surface morphology, consistent with the surface SEM observations.

**Figure 4:**
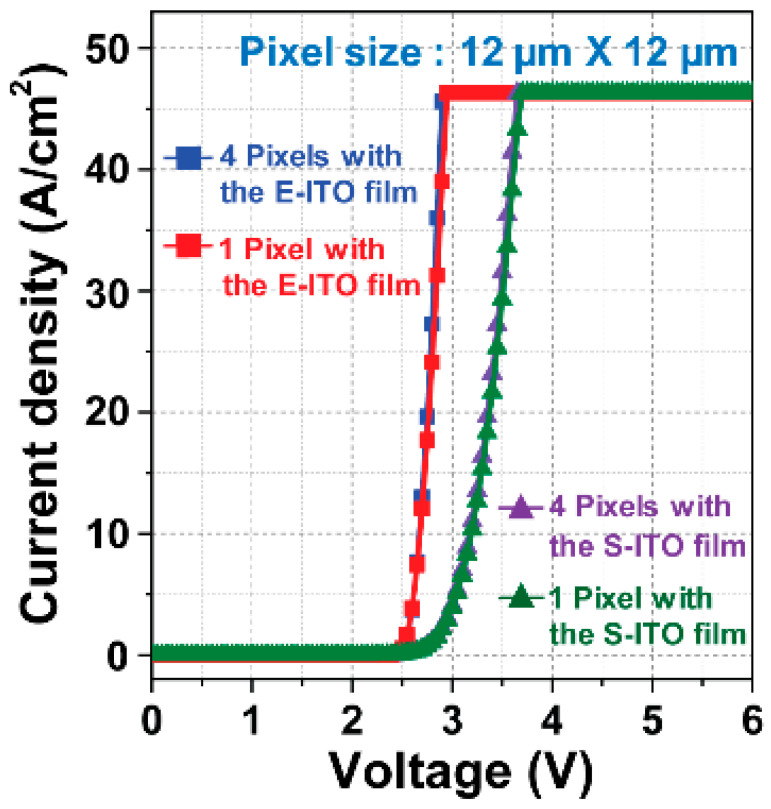
J-V characteristics of blue micro-LEDs for a single pixel and for four pixels with the E-ITO films and S-ITO films.

Figure 4 shows the J-V characteristics of blue micro-LEDs fabricated with the E-ITO films and S-ITO films. The forward voltage (V_F_) values of a single pixel and of four pixels with the E-ITO films were 2.82 and 2.83 V at 30 A/cm^2^, respectively, while those of a single pixel and four pixels with the S-ITO films were 3.50 and 3.52 V at 30 A/cm^2^, respectively. Interestingly, although the S-ITO films exhibited a densely packed morphology and lower resistivity compared to the E-ITO films, the V_F_ values of the micro-LEDs created with the S-ITO films were higher than those of the micro-LEDs created with the E-ITO films. The higher V_F_ values of the S-ITO films could be related as follows: Son et al. reported that the ion bombardment damage introduced by sputtering significantly increased the V_F_ values [36]. Sputtering involves the bombardment of the target material (ITO) with ions, which can damage the underlying layers. In this case, ion bombardment during the sputtering of ITO causes damage to the p-GaN layer in the micro-LED structure. The plasma-induced damage to the p-GaN layer is associated with a notable increase in the V_F_ values, suggesting changes in the electrical properties of the p-GaN layer. Also, Son et al. suggested a physical mechanism for the generation of plasma-induced damage on the p-GaN layer by the plasma electrons [36]. The plasma electrons yielded energetic adatoms of the ITO film on the p-GaN layer during sputtering, through a transfer of energy from the electrons to the adatoms, and increased the plasma-induced damage on the p-GaN layer. In addition, Tian et al. reported that surface damage to the p-GaN layer was specifically attributed to nitrogen vacancies on the p-GaN surface [37]. The sputtering process, during which negative voltage is applied to the ITO target, generates O^2−^ sputtering ions with a greater bombardment effect on the p-GaN crystal surface. This results in the loss of more nitrogen atoms on the p-GaN crystal surface, causing surface damage and potentially altering the electrical characteristics of the p-GaN layer. The presence of nitrogen vacancies at the ITO/p-GaN interface has been identified as a factor contributing to the higher V_F_ values in micro-LEDs with S-ITO films [38]. Consequently, both ion bombardment damage during sputtering and surface damage with nitrogen vacancies on the p-GaN layer could lead to higher V_F_ values in micro-LEDs created with S-ITO films, despite their densely packed morphology and lower resistivity compared to E-ITO films.

**Figure 5:**
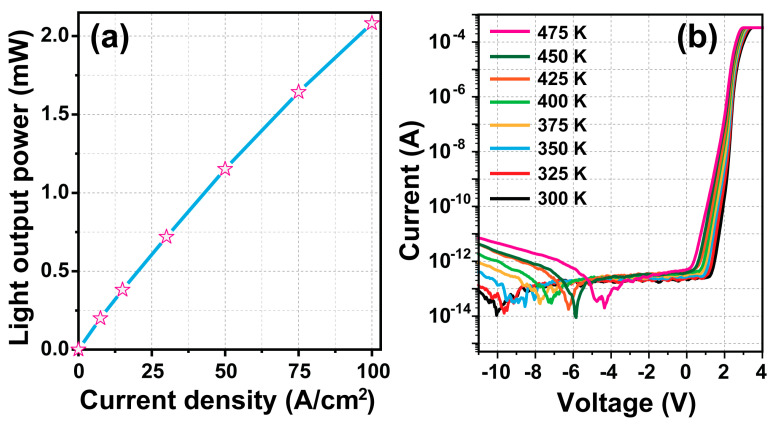
(**a**) Optical output power and (**b**) temperature-dependent current–voltage (T-I-V) characteristics of blue micro-LEDs for 120 pixels using an E-ITO spreading layer.

The optical output power of blue micro-LEDs for 120 pixels was plotted as a function of the injection current density and is shown in Figure 5a. The value of the optical output power for 120 pixels was 1.2 mW at 50 A/cm^2^, and the optical output power was linearly increased when the injection current density was increased from 0.01 to 100 A/cm^2^. One of the important characteristics of blue micro-LEDs is their reliability. For reliability analysis, leakage current characteristics of blue micro-LEDs for 120 pixels were measured as a function of the temperature, as shown in Figure 5b. The reverse and forward currents of blue micro-LEDs gradually increased in the temperature range of 300–475 K. The leakage current can increase due to the increase in point defects either inside the active region or at the mesa-sidewalls. Also, the leakage currents showed a temperature-dependent effect, increasing from 0.1 to 0.8 pA at a reverse voltage of −5 V. However, this is still small enough to be ignored for device operation. It is noteworthy that blue micro-LEDs can function properly under high-temperature conditions. Also, the low device efficiency has spawned great interest in improving micro-LEDs’ performance. High-efficiency micro LED research is worth developing in future works.

**Figure 6:**
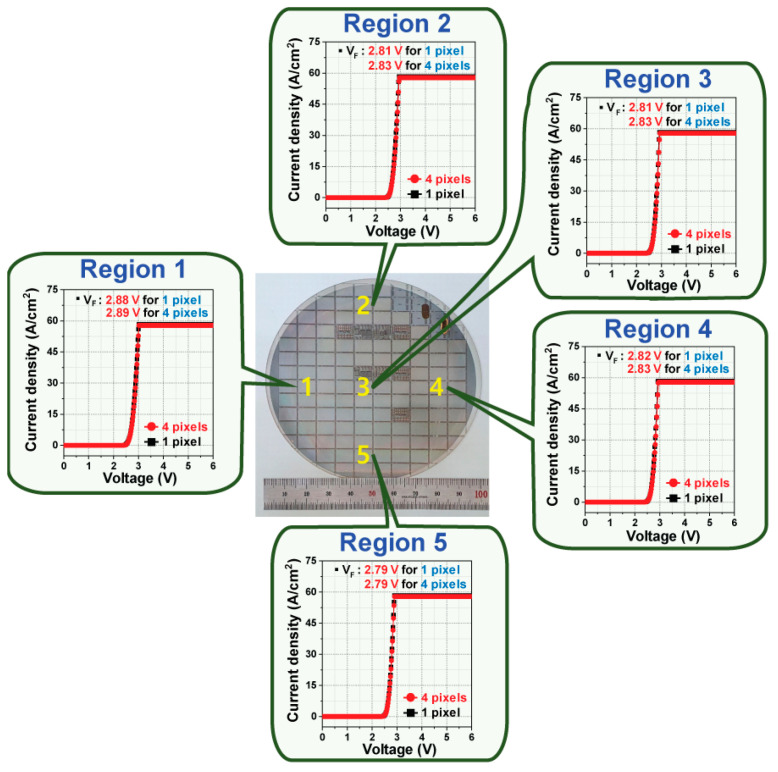
Wafer-scale uniformity of the V_F_ characteristics of blue micro-LED arrays for a single pixel and for four pixels using an E-ITO spreading layer with five different regions.

For industrial mass production, the uniformity of micro-LEDs’ pixel electrical parameters, such as the V_F_ characteristics, is a very important factor for high-quality displays. As shown in Figure 6, the V_F_ values of a single pixel with the optimized E-ITO spreading layer from region 1 to region 5 on a four-inch wafer were 2.88, 2.81, 2.81, 2.82, and 2.79 V at 30 A/cm^2^, respectively. Also, with four pixels, the corresponding V_F_ values of the five different regions were 2.89, 2.83, 2.83, 2.83, and 2.79 V at 30 A/cm^2^. Surprisingly, the V_F_ variations for a single pixel and for four pixels with five different regions were only 0.09 V (3.13%) and 0.10 V (3.46%), respectively. These V_F_ variation values are very low, and they show a narrow distribution on the four-inch wafer. This extremely uniform V_F_ variation is suitable for improving the quality and performance of displays created via industrial mass production.

**Figure 7:**
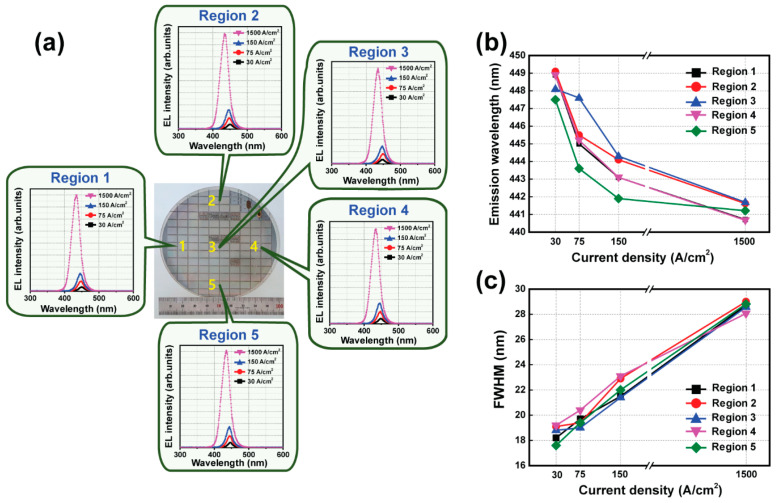
Wafer-scale uniformity of (**a**) the EL spectra, (**b**) the emission wavelength, and (**c**) the FWHM of blue micro-LED arrays for 120 pixels using an E-ITO spreading layer with five different regions on a four-inch wafer as the current density was increased from 30 to 1500 A/cm^2^.

Inefficient micro-LEDs can induce self-heating of the micro-LED device, which can further deteriorate the performance of the micro-LEDs, particularly the emission wavelength. The variation in the emission wavelength of 120 pixels with the optimized E-ITO spreading layer from region 1 to region 5 on a four-inch wafer was measured as the current density was increased from 30 to 1500 A/cm^2^, as shown in Figure 7a,b. The values of the emission wavelength for 120 pixels from region 1 to region 5 on a four-inch wafer were 448.9, 449.1, 448.1, 448.9, and 447.5 nm at 30 A/cm^2^, respectively. The variation in the emission wavelength at 30 A/cm^2^ with five different regions was only 1.6 nm. As the current density was increased from 30 to 1500 A/cm^2^, the blue shifts in the EL peaks’ wavelength were approximately 8.2, 7.5, 6.4, 8.2, and 6.3 nm from region 1 to region 5, respectively. These blue shifts can be attributed to the screen effect on the quantum-confined stark effect (QCSE) and to the enhanced carrier-band filling effect with an increase in the injection current density, during which excess carriers would occupy the higher energy-level states [39,40,41]. In addition, the variation of the full width at half-maximum (FWHM) for 120 pixels with the optimized E-ITO spreading layer from region 1 to region 5 was analyzed, as shown in Figure 7c. As a function of the injection current density, the variation in the FWHM values from regions 1 to 5 was approximately 10.5, 9.9, 9.8, 8.9, and 11.2 nm, respectively. The FWHM of a GaN-based LED can be affected by the quality of the quantum well, the well width, and any indium fluctuations [42]. The emission wavelength and the FWHM of 120 pixels at different injected current densities from region 1 to region 5 are summarized in Table 1. These variations in the emission wavelength and the FWHM were very low, and they showed a narrow distribution on a four-inch wafer. This high-performance blue micro-LED pixel with the optimized E-ITO spreading layer provides a promising and practical solution to achieve micro-LED displays for use in AR and VR applications, circumventing the bottleneck in the development of long-lifespan and high-brightness organic LED devices.

**Figure 8:**
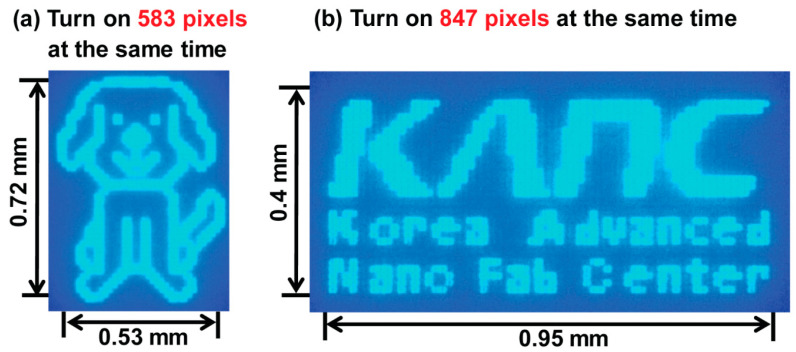
Photographs of zoomed-in images of PM-type blue micro-LEDs with light-emitting images at the same time: (**a**) 583 pixels and (**b**) 847 pixels.

Figure 8 shows the emission images of PM-type blue micro-LEDs created with the optimized E-ITO films. Zoomed-in images of the PM-type blue micro-LEDs showing the light-emitting outcomes at 583 pixels and 847 pixels simultaneously demonstrate good display uniformity and brightness. Optical microscope images of the light emission results highlight the uniformity and confirm the overall excellent image quality, validating the applicability of these micro-LEDs for use in high-performance display technologies. This demonstration emphasizes the tremendous potential of these blue micro-LEDs for display applications requiring high-performance capabilities. This observation has significant implications with regard to the advancement and potential commercial viability of micro-LED displays, indicating their capacity to meet stringent criteria for image quality in practical applications.

## 4. Conclusions

We successfully demonstrated the four-inch wafer-scale fabrication of high-resolution blue micro-LED arrays created using optimized e-beam-deposited and sputter-deposited ITO layers on a sapphire substrate, achieving a density level of 1692 PPI. The surface morphology of the S-ITO films was relatively smooth and dense, while that of the E-ITO films was rather rough. The roughness of the E-ITO films was approximately 7.72 times greater than that of the S-ITO films. Also, the measured resistivity of the S-ITO films was 4.86 × 10^−4^ ohm·cm, much lower than that of the E-ITO films, at 5.96 × 10^−3^ ohm·cm. Interestingly, although the S-ITO films exhibited a densely packed morphology and lower resistivity compared to the E-ITO films, the V_F_ values of a micro-LED created with the S-ITO films were higher than those of a micro-LED created with the E-ITO films. The V_F_ values of a single pixel with the optimized E-ITO layer from region 1 to region 5 on a four-inch wafer were 2.88, 2.81, 2.81, 2.82, and 2.79 V at 30 A/cm^2^, respectively. Also, with four pixels, the corresponding V_F_ values of the five different regions were 2.89, 2.83, 2.83, 2.83, and 2.79 V at 30 A/cm^2^. Surprisingly, the V_F_ variations of a single pixel and of four pixels with five different regions were only 3.13% and 3.46%, respectively. As the current density was increased from 30 to 1500 A/cm^2^, the blue shifts in the EL peaks’ wavelength were approximately 8.2, 7.5, 6.4, 8.2, and 6.3 nm from region 1 to region 5, respectively. In addition, the corresponding variations in the FWHM values on a four-inch wafer were approximately 10.5, 9.9, 9.8, 8.9, and 11.2 nm, respectively. The values of V_F_, the emission wavelength, and the FWHM were very low and showed a narrow distribution on the four-inch wafer. Also, various emission images of PM-type blue micro-LEDs utilizing the optimized E-ITO spreading layer at 583 pixels and 847 pixels simultaneously demonstrated good display uniformity and brightness. The optimal conditions for ITO deposition are important to minimize deposition damage at the interface between the ITO film and the p-GaN layer. The control of the optimal deposition conditions for ITO films is an important way to achieve enhanced performance of blue micro-LEDs. These observations highlight the immense potential of blue micro-LEDs for demanding display applications, showcasing their ability to meet rigorous criteria for superior image quality in practical applications.

## Figures and Tables

**Table 1:** Variations in the emission wavelength and the FWHM of blue micro-LED arrays for 120 pixels with five different regions as the current density was increased from 30 to 1500 A/cm^2^.

Position	EL Property	Current Density			
		30 A/cm^2^	75 A/cm^2^	150 A/cm^2^	1500 A/cm^2^
Region 1	Emission wavelength [nm]	448.9	445.0	443.1	440.7
	FWHM [nm]	18.2	19.7	21.5	28.7
Region 2	Emission wavelength [nm]	449.1	445.5	444.1	441.6
	FWHM [nm]	19.1	19.4	22.9	29.0
Region 3	Emission wavelength [nm]	448.1	447.6	444.3	441.7
	FWHM [nm]	18.8	19.0	21.4	28.6
Region 4	Emission wavelength [nm]	448.9	445.2	443.1	440.7
	FWHM [nm]	19.2	20.4	23.1	28.1
Region 5	Emission wavelength [nm]	447.5	443.6	441.9	441.2
	FWHM [nm]	17.6	19.4	22.0	28.8

## Data Availability

The original contributions presented in the study are included in the article, further inquiries can be directed to the corresponding author.
